# A primary mediastinal germ cell tumor of yolk sac type: case report

**DOI:** 10.11604/pamj.2021.38.330.23730

**Published:** 2021-04-06

**Authors:** Khadija Darif, Zineb Benbrahim, Nisrine Acharfi, Anass Khacha, Mustapha Maaroufi, Lamiae Amaadour, Karima Oualla, Samia Arifi, Nawfel Mellas

**Affiliations:** 1 Department of Medical Oncology, Hassan II University Hospital, Fez, Morocco,; 2 Faculty of Medicine and Pharmacy of Fez, Sidi Mohamed Ben Abdellah University, Fez, Morocco,; 3 Department of Radiology, Hassan II University Hospital, Fez, Morocco

**Keywords:** Germ cell tumors, yolk sac tumor, mediastinum, case report

## Abstract

The mediastinal malignant germ cells tumor represents less than 0.5% of thoracic tumors, although the mediastinum is one of the main extragonadic locations of these tumors. In the majority of cases, young people are those most affected. The prognosis of mediastinal malignant germ cells tumors is poor, especially non-seminomatous germ tumors. In this article, we report a rare case of a young 19-years-old patient treated for a mediastinal germ cell tumor of yolk sac. The patient presented a chest pain; the chest computed tomography (CT) showed a right paramedian mediastinal mass with a pleural effusion associated with supraclavicular and cervical lymph nodes. Biopsy revealed a non-seminomatousgerm cell tumor of yolk sac. The exams showed elevated alpha-fetoprotein (AFP), without any meaningful elevation of other serictumor markers. The patient received 4 cycles of chemotherapy based on etoposide, ifosfamide and platinum salts then a complete excision of the mass.

## Introduction

Germ cell tumors (GCT) are rare tumors that arise from primordial germ cells. They are classified into two main categories: seminomas and non-seminomas and are usually located in gonadal sites (testis in men) [1]. The yolk sac tumors, also called endodermal sinus tumours, are non-seminomatous malignant GCTs that are histologically similar to the yolk sac and extraembryonic mesenchyme. They have been described for the first time by Teilmann *et al*. These tumors produce significantly alpha-fetoprotein (AFP) [1, 2] and are usually associated with poor prognosis despite the existence of the modern chemotherapy. In this paper, we report a case of a young patient with a primary mediastinal yolk sac.

## Patient and observation

**Figure 1:**
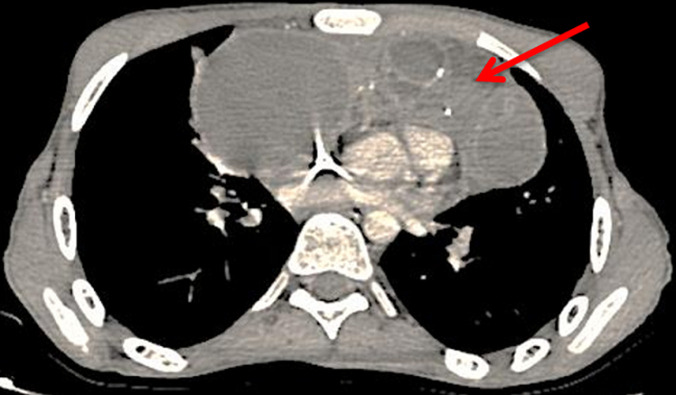
chest computed tomography scan revealing a mediastinal mass

**Figure 2:**
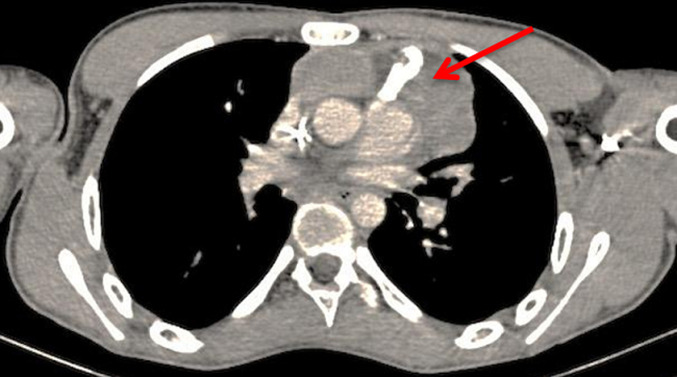
chest computed tomography scan after chemotherapy demonstrating a partial response

We report the case of a 19 years old patient who presented at the Hassan II University Hospital with a 3 months history of chest pain and dyspnea. The initial clinical examination revealed a slight reduction in vesicular murmurs in the right lung field, and a left cervical lymph node. The X-ray thoracic radiography revealed a non-compressive tracheo-bronchial opacity with para-hilar calcification. Chest tomography (CT) scan revealed a voluminous right paramedian mass measuring 170 mm of diameter, with significant central calcification and infiltration of the pulmonary aorta and left pulmonary parenchyme; the left subclavicular lymphe nodes measuring 25 mm and a medium pericardial effusion were also found (Figure 1, Figure 2).The biopsy of the cervical lymph node revealed a poorly differentiated malignant tumor corresponding to a non-teratomatous germ cell tumor of yolk sac type, the immunohistochemistry confirmed the histological diagnosis with positive staining on PALP, AFP and pancytokeratine antibodies. The testicular ultrasound, abdominal CT scan and bone scan, did not reveal any anomalies. The serum level of alpha-fetoprotein was elevated to 23224ng/ml whereas ß-hCG was within the normal range.

The patient received 4 cycles of chemotherapy based (VIP): Etoposide (75mg/m^2^ days 1 to 5), Ifosfamide (1500 mg/m^2^ days 1 to 5), Uromitexan (1500mg/m^2^ days 1 to 5) and Cisplatin (20mg/m^2^ days 1 to 5) with 480 micrograms subcutaneous daily injection of filgrastim during 5 consecutive days. Biological assessment revealed an AFP decrease. Radiological evaluation objective a partial response according to the Response Evaluation Criteria in Solid Tumours (RECIST) version 1.1. A residual mass measuring 100mm has been shown. The patient underwent a surgical intervention, based on complete excision of all residual anterior mediastinal tumors with resection of the phrenic nerve. The anatomopathological examination revealed a total and massive necrosis of the tumor with contingent of mature teratomas with no viable cells. The patient is in complete biological and radiological remission without any evidence of recurrence after 42 months of follow up.

## Discussion

Germ cell tumors (GCTs) are classified as extragonadal if there is no evidence of a primary tumor in the testes. These tumors include seminomas, non-seminomatous GCTs, mature teratomas, and immature teratomas based upon histology [3]. About 20% of Yolk sac tumors (YSTs) arise in extragonadal sites, including the mediastinum, sacrococcygeal region, cervix, vulva, pelvis, liver, prostate, and retroperitoneum [4, 5]. YSTs can occur in both men and women, usually arising from germ cells in testes and ovaries, respectively. Pure YST tumors are usually found in young children, whereas mixed germ cell tumors with YST are found in adults [6].

Histologically, the histogenesis of extragonadal YST is not well defined, several proposed hypotheses can explain the existence of germ cell tumors in extragonadal sites [7, 8], in our case the stop of the migration of germ cells through the mediastinum could explain the occurrence in this site. Other hypotheses can be mentioned, such as an aberrant differentiation of somatic cells, or the presence of an occult focus metastasis in the testis.

Clinically, mediastinal germ tumors usually manifest as a dyspnea (25% of cases) with chest pain (23% of cases). Other described symptoms include, according to an international study reporting 381 mediastinal GCTs, fever (13%), night sweats or weight loss (11%). Fatigue, hemoptysis and superior vena cava syndrome were seen in less than 10% of patients with mediastinal GCT [9]. Similarly to other non-seminomatous germ tumors, YSTs can be associated with haematological Klinefelter´s syndrome and other hematological malignancies such as acute leukemia and myelodysplastic syndrome.

On the biological level, high rates of AFP are usually objective in the YSTs at the moment of diagnosis and they significantly decrease after treatment. Thus, it is considered as a predictive marker for therapeutic response. Similarly to extragonadic non seminomatous GCTs, mediastinal YSTs carry a poor prognosis with 40-50% overall survival and puts this entity in the poor prognosis group according to the International Germ Cell Cancer Collaborative Group (IGCCCG). Systemic treatment of YSTs is based on the same chemotherapy regimens that are used for patients with advanced, intermediate- or poor-risk gonadic GCTs. However, VIP is preferred, rather than BEP regimen for the mediastinal sites.

Actually mediastinal GCTs are typically candidates to thoracotomy to excise the residual disease. This may require prolonged exposure to high partial pressures of oxygen during surgery and lead to bleomycin-related pneumonitis. Indeed, in a retrospective study reporting 221 patients who underwent thoracic surgery, 13% of patients treated with BEP developed acute respiratory distress syndrome versus 0% with VIP. There were 6.6% of post operative death with BEP mainly related to pulmonary failure, versus no deaths in those treated with VIP [10]. After completing systemic chemotherapy, residual mediastinal masses may persist. Resection of all remaining lesions masses after chemotherapy should be performed whenever it is technically feasible. Following postchemotherapy surgery, no further therapy is necessary in case of complete pathological response. If viable cells are identified, two additional cycles of VIP chemotherapy should be given.

## Conclusion

The case presented in this paper shows the importance of multidisciplinary approach which offers the best chance of survival to these patients; in our case the combination of chemotherapy and surgery with complete exercise resulted in complete remission of the disease, our patient achieved a 42-month recurrence-free survival.
